# Oncologists’ reflections on patient rights and access to compassionate use drugs: A qualitative interview study from an academic cancer center

**DOI:** 10.1371/journal.pone.0261478

**Published:** 2021-12-17

**Authors:** Jeremiah Stout, Cambray Smith, Jan Buckner, Alex A. Adjei, Mark Wentworth, Jon C. Tilburt, Zubin Master

**Affiliations:** 1 Mayo Clinic Alix School of Medicine, Rochester, MN, United States of America; 2 Biomedical Ethics Research Program, Mayo Clinic, Rochester, MN, United States of America; 3 UNC Chapel Hill School of Medicine, Chapel-Hill, NC, United States of America; 4 Department of Oncology, Mayo Clinic, Rochester, MN, United States of America; 5 Digestive Disease and Surgery Institute, Cleveland Clinic, Cleveland, OH, United States of America; 6 General Internal Medicine, Mayo Clinic, Scottsdale, AZ, United States of America; 7 Center for Regenerative Medicine, Mayo Clinic, Rochester, MN, United States of America; Deakin University, AUSTRALIA

## Abstract

The U.S. Food and Drug Administration (FDA) allows patients with serious illnesses to access investigational drugs for “compassionate use” outside of clinical trials through expanded access (EA) Programs. The federal Right-to-Try Act created an additional pathway for non-trial access to experimental drugs without institutional review board or FDA approval. This removal of oversight amplifies the responsibility of physicians, but little is known about the role of practicing physicians in non-trial access to investigational drugs. We undertook semi-structured interviews to capture the experiences and opinions of 21 oncologists all with previous EA experience at a major cancer center. We found five main themes. Participants with greater EA experience reported less difficulty accessing drugs through the myriad of administrative processes and drug company reluctance to provide investigational products while newcomers reported administrative hurdles. Oncologists outlined several rationales patients offered when seeking investigational drugs, including those with stronger health literacy and a good scientific rationale versus others who remained skeptical of conventional medicine. Participants reported that most patients had realistic expectations while some had unrealistic optimism. Given the diverse reasons patients sought investigational drugs, four factors—scientific rationale, risk-benefit ratio, functional status of the patient, and patient motivation—influenced oncologists’ decisions to request compassionate use drugs. Physicians struggled with a “right-to-try” framing of patient access to experimental drugs, noting instead their own responsibility to protect patients’ best interest in the uncertain and risky process of off-protocol access. This study highlights the willingness of oncologists at a major cancer center to pursue non-trial access to experimental treatments for patients while also shedding light on the factors they use when considering such treatment. Our data reveal discrepancies between physicians’ sense of patients’ expectations and their own internal sense of professional obligation to shepherd a safe process for patients at a vulnerable point in their care.

## Introduction

Since the 1970s, the U.S. Food and Drug Administration (FDA) has allowed patients with serious diseases who have exhausted other options to access unapproved investigational drugs outside of the clinical trial setting [1]. This process has historically been facilitated by the FDA’s Expanded Access Program, which was first formalized in the late 1980s. Expanded access (EA) can be used for single patients (also known as “compassionate use”), intermediate size populations, or treatment group populations all of which require FDA and ethics review by an institutional review board (IRB). Single patient compassionate use requests can be made for emergency or non-emergency situations and response times average less than 1 day for emergency requests [1]. More than 99% of all requests are approved, and the FDA has taken recent steps to reduce the application to two pages and to provide assistance for the process [1–3].

Despite these efforts by the FDA, 41 states have passed new “Right to Try” (RTT) laws since 2014 [4]. This right to try movement has been led by the Goldwater Institute, a libertarian public policy organization that has advocated for the adoption of “right to try” laws that would reduce FDA powers and oversight from the process of approving non-trial use of experimental medication [5]. RTT laws however do not mandate companies to provide experimental medication to patients and thus fall short of their name and intent. Despite this issue, RTT laws are favored by the public and members of congress on both sides of the aisle. In May 2018, congress passed the federal RTT Act, which created another federal pathway for terminally ill patients to access treatments that had passed Phase I clinical testing without requiring FDA and IRB oversight.

The growing right to try movement, and the passage of the federal RTT law, have brought new scrutiny to the complex ethics of providing experimental medications outside of clinical trials [6]. Access is typically justified ethically by compassion for individual patients with terminal illnesses and respect for their autonomy [7, 8]. However, these goods must always be weighed against patient safety, the long term value of regulatory structures, and the societal benefit of clinical trials to public health [9–11]. The difficulty of balancing safety and public health on the one hand with timely and equitable access on the other [12, 13] makes clear the need to develop robust guidance for patients and clinicians navigating this landscape [14, 15].

The COVID-19 pandemic has made the tension between the desire for rapid access and the need for strong empirical evidence of safety and efficacy through clinical trials more apparent and urgent [16–18]. Questions have been raised about the complex ethics of providing drugs with unknown safety and efficacy data during a pandemic [19, 20], the appropriateness of different regulatory mechanisms in overseeing unapproved treatments for the public [16, 21, 22], and the professional obligations of physicians in utilizing preapproval mechanisms [23].

The 2018 RTT law’s removal of required IRB and FDA oversight places more responsibility on requesting physicians, but little empirical work has explored how physicians perceive their role and responsibilities in non-trial access to investigational drugs [1, 24–26]. Oncology and hematology physicians are among the most frequent prescribers of compassionate use drugs [27, 28]. Understanding how oncologists with compassionate use experience interpret the approval process and navigate its challenges may clarify the practice implications of new policy changes and lend insights to the kinds of guidance clinicians need for existing pathways to be beneficial to their patients. Given that oncologists, even those with experience providing off-trial medication, can conflate definitions, policies and procedures between compassionate use, RTT and off-label [26], capturing their understanding of and experiences with different preapproval pathways is important if we are to design education to inform and aid physicians with varying patient requests for experimental medications. In this study, we interviewed oncologists from an academic medical center with three major geographic sites across the U.S. who had compassionate use experience to understand their experiences, knowledge, and opinions of the approval processes and pathways.

## Methods

We conducted semi-structured interviews to capture the views and experiences of academic oncologists at a major cancer center with access to federal RTT and EA programs, the methods of which have been previously reported [26]. This study was designated minimal risk and a waiver of signed consent was approved by the Mayo Clinic Institutional Review Board #19–005556. Prior to obtaining informed consent, all subjects were provided information regarding the study goals, risks and benefits, procedures used including the interview process, recording and transcription, methods to protect privacy including deidentification, deletion of recordings and maintaining confidentiality in reporting data, and procedures for withdrawal. All subjects provided verbal consent to participate which was documented by the interviewer.

### Recruitment

The Mayo Clinic Cancer Center is designated a comprehensive cancer center by the National Cancer Institute with locations in Arizona, Florida, and Minnesota. The Cancer Center examines a full spectrum of multidisciplinary cancer research with 360 physicians and scientists across 43 departments and provides comprehensive whole-person cancer care. With numerous active clinical trials, many oncologists provide standard of care treatments and will enroll eligible and willing patients in clinical trials and EA. Additionally, there is administrative support for most oncology specialities with processing EA requests.

We used the Mayo Clinic Investigational Drugs-Devices Database to identify a convenience sample of oncologists at Mayo Clinic who had experience with EA from 2014–2019. At the time the study was conducted, Mayo Clinic had approximately 155 clinical oncologists among its three campuses located in the southwest, midwest, and southeast areas of the country. We sent email invitations to all 39 oncologists in the database to participate in an interview study and sent up to two follow up email invitations to those who did not reply. We identified additional eligible participants who did not appear in the database by snowball sampling and by sending an email invitation to all oncologists at Mayo Clinic. All participants were screened to ensure alignment with eligibility criteria.

### Interview guide development

Our initial interview guide was developed *a priori* after a review of the academic literature on compassionate use ethics, policy, and stakeholder perceptions. Noting a lack of knowledge of RTT among our initial set of interviews (n = 3), we revised our interview guide to include a brief introduction to the federal law and its provisions. The final interview guide was divided into two parts: a brief screening and self-assessment to gauge participants’ experience and familiarity with EA and RTT, and 25 questions covering 7 areas: demographics; knowledge and familiarity with EA; physician-patient conversations about investigational drug use; consenting patients; experience with EA; familiarity with RTT; and ethical and professional obligations under EA and RTT. The interview guide has been published as supplemental information previously [26] and is also found in S1 Interview guide.

### Data collection and analysis

The interviews were conducted either in person or by telephone from October 2019 to February 2020 and concluded when thematic saturation was reached at 21 interviews. The interviewers (CS and ZM) followed the guide described above and provided additional clarifications and definitions when requested but refrained from answering questions about the political history of the law that might bias participants’ responses to questions later in the interview. All interviews were audio-recorded and transcribed for analysis.

Qualitative analysis of the interview was performed using modified grounded theory [29] with constant comparison analysis [30]. An initial codebook was drafted after review of ten transcripts by two analysts (JS and CS). This codebook was updated after in-depth coding of three additional interviews and the final codebook was used for all 21 interviews. Each transcript was coded in duplicate by both coders (JS and CS) and discrepancies were resolved by discussion.

Our study is derived from a single dataset where we examined oncologists’ knowledge, experiences and attitudes towards RTT (originally published in Smith et al., 2021 [26]), expanded access, and oncologists’ reflections towards physician obligations in offering off-trial medication to patients. We divided the results to prevent reporting a large and cumbersome dataset in a single manuscript. This practice aligns with the Consolidated Criteria for Reporting Qualitative Research [31]. The results reported here reflect our analysis of participants’ knowledge, experiences and attitudes with EA (compassionate use) requests and their broader reflections on physicians’ professional and ethical obligations to patients who request off-trial access to unapproved medications. The analyses and reporting are distinct and there is no overlap in the results reported, including any repetition of quotations.

## Results

Twenty-one medical oncologists from Mayo Clinic in Minnesota, Arizona or Florida participated in our study, representing several oncology sub-specialties. Participants had practiced medicine for 17 years on average (7 to 34 years range) at the time of interviews, and most oncologists had research and administrative duties in addition to their clinical responsibilities. We report five major themes that emerged from our analysis examining oncologists’ attitudes towards EA and reflection on physician obligations to provide off-trial access (Table 1).

**Table 1:** Summary of themes.

**Theme 1: Ease with Experience**
Oncologists had mixed reviews on the ease of accessing experimental drugs through EA and that past experience was likely to have eased administrative burdens.
**Theme 2: Science or skeptic**
Oncologists reported differing rationales of patients considering experimental interventions where most patients had a strong science and health understanding of the intervention while others were skeptical of conventional medicine and interested in pursuing natural or alternative options.
**Theme 3 Enthusiasts or Realists**
Oncologists reported that the majority of patients had realistic expectations of experimental medicine i.e., improving quality of life, while some had unrealistic optimism expecting a miracle cure.
**Theme 4: No “Right” to Try**
Oncologists reported that terminally ill patients can ask for any experimental drug but should not have a right to try any drug they desire because some options are unreasonable and can cause harm.
**Theme 5: An Oncologist’s Duty to Seek Compassionate Use Drugs for the Right Patient**
Oncologists reported that it is their professional obligation to seek experimental drugs through compassionate use for patients who can benefit from them.

### Experiences and considerations for navigating preapproval access to investigational drugs

All oncologists we interviewed had experience navigating compassionate use pathways. Most participants had pursued the pathway for single patient use and some also had experiences enrolling patients in intermediate size EA programs. While several physicians had considered RTT requests, none had requested access to investigational drugs from pharmaceutical companies through this regulatory pathway (see Smith et al. [26] for more details on oncologists’ experiences with RTT).

Nearly all of our participants reported they had raised the possibility of pursuing non-trial access for patients that they thought would benefit. Many oncologists also reported that in one third to one half of cases, the patient was the one who had asked about pursuing an investigational drug. Some participants reported rare cases in which their patients had requested a particular, non-trial pathway such as compassionate use or RTT. In most cases, however, their patients who requested experimental therapies did not specify whether they wanted to enroll in a trial or use non-trial pathways to gain access to those drugs.

Outcomes of the process were mixed for our participants. Even though all of our participants had navigated the process, some had not administered the drugs because their requests were denied by companies or the IRB, their patients died before they could get access to the drug, the drug was approved before their request was completed, or their patient did not yet need the drug they had requested. Among those who had administered the drug, there were a handful of participants who saw dramatic responses from patients. The majority, however, saw moderate, little, or no benefit for their patients. Regardless of the outcomes, most physicians were willing to pursue EA again, although that willingness was often contingent on the presence of administrative support.

#### Ease with experience: Mixed reviews on the ease of accessing experimental drugs through EA

Our participants gave mixed reports on the ease of accessing investigational drugs falling in two main groups. One group reported that non-trial access was difficult to obtain due to the complexity of the process and unwillingness of sponsors to make the drug available. The other group reported that it was not difficult for them to gain access and that companies were usually receptive. Some who had success in the past acknowledged that the process is much easier once a physician has been through it, noting they could imagine it being much more difficult now than it was in their particular experience.

It’s not easy at all in my opinion for a number of reasons. One, [companies] actually have to have a willingness and an access program for you to be able to get access to it. Two, you’ve got to make sure that it’s the right thing for your patient. (Participant 15)

I haven’t noticed any significant barriers because I’ve tried only three or four times with two companies and both of them, like I told you, the people on the other side have been really responsive. They just want to make sure we have a good rationale and [that]. . .the patient fits most of the clinical trial-ish criteria that they have set up before. Other than that, I haven’t found much difficulty. (Participant 16)

Regardless of oncologists’ ease with past experiences, there was widespread agreement that the likelihood of gaining access to investigational agents was significantly increased by requesting a drug with enough data to suggest a high likelihood of benefit and applying the drug appropriately to a patient’s specific tumor. Participants also reported that the ease of access to drugs is ultimately determined by the receptiveness and policies of each drug company, and that larger companies were typically easier to work with. Participants also mentioned that other variables, such as insurance coverage and travel to medical centers, may also affect a patient’s ability to use a drug even if it has been made available by the manufacturer.

Oncologists named multiple factors that had played a role in their previous requests (Table 2). First and foremost, participants specified that for a request to be reasonable it must have a strong scientific rationale—typically a genetic mutation or other biomarker offering plausibility of benefit. They also required that the known risks of the drug not outweigh the benefits, that the patient is healthy enough to tolerate possible side effects, and that there are not any other options available to the patient. Participants reported they would be unwilling to consider non-trial access to investigational drugs if there was insufficient safety data and low likelihood of benefit, or if the patient had a poor functional status and was simply taking a “shot in the dark.”

**Table 2:** Oncologists’ reasons for considering non-trial preapproval access to investigational drugs.

**Scientific Rationale and Evidence of Benefit**
I have another patient where she was on hospice. There was a drug and I was able to get the drug because it was for a certain targeted mutation. I knew it was a homerun drug. I gave it to the patient. Now we’re a year later and she got out of hospice. She’s alive, doing really well. (Participant 1)
Most of the time I’m not looking for something that’s like a nebulous phase-one trial. Usually I’m looking for things that are open in phase two elsewhere that we just don’t have available here, or things that maybe have looked really promising in a phase two or phase three trial but have not been FDA approved, so it’ll be hard for us to push that through insurance. (Participant 11)
I do research on [*NAMED CLASS OF DRUG*], so I knew that this is a very powerful drug and could be life-changing for this patient. It wasn’t a drug—at the time, it wasn’t a drug that might give you maybe one or two months of longer life. This was a major drug that could’ve extended life by a year or more in clinical trials. It was a potentially life-changing drug—no way to get it to patients at that time unless it was on a clinical trial, so that’s the reason why I went through the effort. (Participant 12)
**Safety and Functional Status**
I thought about it and I really thought about it. I did not think it was ethical. I thought that it was not—the safety wasn’t established. The patient’s clinical status was really poor. I would never have considered giving her therapy if we weren’t already going down this road. Ultimately, I decided to cancel the medication. (Participant 1)
I didn’t do it because that patient was advanced, a poor performance status, would not be able to tolerate, his lab studies were abnormal, and so that was never an option for that patient. (Participant 15)
The other patient, the drug is part of a class of drugs that’s been under investigation for a couple of years now and have shown a number of results already, and have class of effects that have been mirrored across different agents so that the clinical side effect profile is pretty well known, and it’s pretty well tolerated, so she wasn’t hesitant to go ahead with it. (Participant 8)
**Rare Disease and Lack of Options**
I think in those situations I’ve told the patient I will reach out to the company and see if I can first and foremost always look for clinical trials first. Th[e] first priority is to gain access to these meds through clinical trials. In the most recent case the patient actually screen failed for a really stupid reason, and so that’s why I reached out to the company to say, “Can we treat her off study?” (Participant 10)
These patients that I referred to have extremely rare diseases and extremely poor survival, and the options that we have on the market, none of them work great for them. (Participant 16)
Both patients have [name of tumor type], which doesn’t really have a lot of other options from a treatment standpoint, so I think the less options you have from a cancer treatment perspective, the more willing you are to go out on a limb and try other stuff. (Participant 8)
**Patient Motivation**
It’s not something I typically do unless I really think that there’s a strong reason to do it or a patient’s really pushing for it. (Participant 17)
Again, it also depends on what the patient’s experience has been with previous treatments. If they felt miserable and not having quality of life, then they may not wanna do anything more. If they felt good with whatever treatments they had, they wanna do more, so I think it just depends on the patient’s experience. (Participant 4)
Well, there’re several calculations that go into this. One is what’s the likely potential for benefit. The second one is what’s the experience with the drug and that is, is it reasonably tolerated. The third is what’s the availability to get the drug or a reasonable surrogate outside of this mechanism. Then what’s the patient think? There are some people that are in go mode and it’s like, ‘Yeah. Even though it’s not on the market yet, yeah, I really want it.’ All those things weigh into the calculation, I think. (Participant 21)

### Oncologists’ experiences engaging patients requesting compassionate use drugs

Physicians who brought up or recommended experimental compassionate use options for their patients started from the assumption that a particular drug was a reasonable option for their patient. However, when patients requested specific experimental options, oncologists reported that there was a large amount of variation regarding the “reasonableness” of the request.

Occasionally, patients will be well informed and make very reasonable requests. I’d say maybe 25 percent of the time patients when they request access, have done their homework and have a very reasonable thought process. (Participant 5)

#### Science or skeptic: Differing rationales among patients requesting experimental therapies

Patients who brought up specific compassionate use requests were generally described as motivated and well-connected to potential avenues for additional treatments. Out of that subset of patients, some had a strong grasp of the scientific literature, were highly health literate, and could recognize plausible treatment modalities potentially appropriate for their condition. Oncologists described other patients as skeptical of conventional medicine and hoping for something different from what they had previously been prescribed. Multiple oncologists also described patients who requested specific experimental therapies as committed to more “natural” health options or opposed to any standard-of-care therapies, including refusing chemotherapy.

Actually now that I think about it, she may be the first patient… who wanted CAR-T for her curable disease with drugs that are FDA-approved and have an 80 percent, 70 to 80 percent, response rate for cure, and she wants CAR-T instead. I’m like, ‘Oh, my gosh.’ (Participant 10)

Participants also differentiated between patients based on age, especially when there were young patients suffering with difficult diagnoses. Some physicians reported being more motivated to pursue extra options for these patients, not only because they may have a better functional status than older patients, but because families were also more motivated to pursue experimental therapeutics.

#### Enthusiasts and realists: Patients can have high hopes or realistic expectations

Oncologists reported a range of expectations among patients looking for compassionate use drugs. Some reported that they thought their patients were clearly looking for a miracle or a cure, but others explained that their patients knew that remission was unlikely, and that they simply wanted to try something to help prolong their time or help with quality of life. The majority of oncologists reported that they thought their patients had realistic expectations and a good understanding of the experimental nature of the drug while also having hope that it would improve their condition and/or quality of life. This was especially common when physicians were the ones who brought up the possibility of pursuing the drug through an EA route.

A few oncologists reported that some of their patients had unrealistic optimism and a strong belief that the experimental drug would cure them. Family members were often reported to be the ones who had less realistic expectations than patients. Physicians described patients and families who were fixated on getting a particular drug as less understanding of the experimental nature of the drug and more likely to believe that the drug would cure them or a loved one.

The one that I did not end up giving it to, I don’t think they really understood [the experimental nature of the drug]. I don’t think they really cared…They just wanted the drug. I could have said that it had a zero chance of working and they would have I still think said, ‘I want to try the drug.’ (Participant 1)

Many participants reported wanting to ensure that their patients had realistic expectations before moving forward with the request, but also acknowledged the difficulty of balancing hope and realism in these clinical situations. Some related to this broader practice and explained that as oncologists, they face these sorts of tensions every day with their patients.

Most of the patients where, if I’m gonna move forward with an IND [Investigational New Drug], I would want them to feel similar. Have that hope, but also have that understanding that this might not be that magical pill, because I don’t want to ever mislead patients. I do want them to have hope that there’s a chance, and treatment is going to improve their outcome. I want them to have some understanding, in their own words, that this is a reasonable option. Hopefully, this is what will work, but we also are accepting some risk in doing that. (Participant 11)

Because this is a time-intensive process, physicians also reported using the amount of patient or family motivation as one additional consideration when determining whether to pursue experimental drugs. A few physicians reported initially considering even unreasonable requests from patients due to patient or family insistence, but due to additional knowledge about the drug’s toxicity or lack of supportive data, regulatory guidance, patient deterioration, and/or ethical qualms going forward with a drug unlikely to help a patient, most of these processes stopped before the patient was given the drug. A few physicians reported that this unwillingness to pursue access to the drug fractured their clinical relationships with patients, ultimately ending their therapeutic alliance.

### Oncologists’ professional obligations to consider experimental drugs for their patients

The majority of participants reported that in general, compassionate use drugs were used as palliative care for patients. While a few oncologists described experiences with experimental drugs that resulted in remission or other remarkable clinical outcomes, these situations were rare. Oncologists explained that drugs offered on a compassionate basis largely served to help patients make it to a specific milestone, ease symptom burden, or improve quality of life. Physicians reported wanting to be hopeful about the possibility of a major impact and did have some “home run” cases, but many others had disappointing outcomes from the experimental drugs.

If a patient is using expanded access or compassionate use or right-to-try, an oncologist at that point is not trying to cure. An oncologist, at that point, is not trying to save that life. The oncologist, at that point, is really just trying to buy more time because, by the time a patient needs those services, they will have already been through the best drugs that we have for that cancer. You cannot expect a drug that has no proven efficacy to be better than our standard of care. That’s the reason why—if people are using expanded access or right-to-try because they think they’re going to get cured, they will have a very big disappointing outcome because that is not our goal as oncologists. (Participant 12)

#### No “right” to try: Patients should not be able to try any drug they wish

We also asked participants whether terminally ill patients should have a right to try investigational drugs. All but one of our participants agreed that terminally ill patients should not be allowed to try any drug they wish. Several participants suggested that because a physician’s knowledge and guidance is necessary for patients to access investigational drugs, patients should have a right to ask for experimental therapies or discuss them with their oncologist instead of a right to try any drug they wish. Our participants explained that because most patients do not have the medical understanding to know which drugs are reasonable options, it is essential that physicians play a role in determining whether or not there is a good rationale for attempting to use an investigational drug.

I don’t think most of my patients have the medical education to make that decision. I mean, they come in saying, ‘We read about this trial; we want this drug.’ I said, ‘Well, that’s a drug for leukemia. That is unlikely to do anything meaningful for your lung cancer.’ (Participant 9)

Several participants mentioned that with increased access to health information online, there were both opportunities and challenges for engagement with patients. With increased access to information about their disease, patients may be able to find reasonable opportunities to explore, but physicians also reported that patients often found false hope or unrealistic options online.

[When a patient brings up an experimental therapy], there are sometimes where it’s a very valid question. Other times it’s just like they’re trying to search for an answer. It’s an emotional need that’s unaddressed. You talk about, ‘Okay, how are you coping with this?’ Sometimes it’s a coping mechanism. They’re up at 3:00 a.m. They can’t sleep, and they’re trying to find the answer. They’ll believe what anybody ever tells them on Google. It’s a challenge. We want patients to be informed and read up on the disease and be familiar with it, but in the same token, if someone’s looking for unfounded hope, it’s out there if they want to find it. (Participant 17)

#### An oncologist’s duty to seek compassionate use drugs for the right patient

Many oncologists described the effort of accessing investigational drugs as above and beyond typical clinical duties but also considered it to be part of their professional obligations for patients that could potentially benefit from an experimental drug.

Personally, I see that as part of the job… it’s not above and beyond, but the effort is above and beyond because we get no clinic time to do these things. (Participant 12)

It’s a time sink and that’s it. Apart from that, if it’s the right thing to do, you spend the time. (Participant 6)

I think it’s just something I do when I think it may help the patients and it’s obviously part of my responsibility. (Participant 5)

Most specified that this is a duty more relevant for academic clinicians, since they perceived their job to include keeping up with promising experimental options. Given this, however, many clarified that compassionate use is only appropriate in rare clinical cases. Some also explained that it must be coupled with patient education and clear expectations that these drugs very rarely cure. Many participants stated that they understood why other physicians chose not to pursue this avenue for patients given the amount of time and energy it required, but in their particular circumstances they expected themselves to help patients navigate access to reasonable drugs.

## Discussion

The passage of the federal RTT and the recent COVID-19 pandemic have reignited questions about the ethics of using medications without known safety and efficacy, but little is known about physicians’ experiences with and attitudes toward existing pathways that could help guide the development of future policy [25, 26, 32]. The diversity of views and experiences in discussing and requesting experimental drugs through EA in our study provide additional clinical perspectives on patient access to unapproved investigational drugs outside of clinical trials.

While institutional support was available for oncologists in our study to access experimental drugs via EA, several oncologists reported challenges in navigating the multi-stakeholder and multi-administrative processes similar to the experiences of physicians captured in other studies [1, 32]. The biggest barriers our participants identified were the navigation of complex websites and forms, and the receptiveness of drug companies to requests for EA. Institutions can do their part to help streamline administrative processes by providing a centralized process and identifiable experts to help physicians navigate FDA, IRB, and potentially other institutional processes. Additionally, a database like the one used in this study to recruit oncologists could improve access by identifying different drugs, patient conditions, and the indications for use among EA requests to minimize duplicative efforts. Despite administrative hurdles, oncologists in our study were willing to use EA again. Similar attitudes were found in an FDA commissioned study where 94% of 139 physicians reported a willingness to recommend EA programs to a colleague despite difficulties they encountered [1]. This is further echoed by the finding that our cohort of oncologists reported that accessing investigational products was part of their professional obligation to patient care but remained cognizant of the reasons why other clinicians might choose not to offer EA to patients, especially physicians at institutions that do not provide the support they perceived to be necessary to navigate EA pathways.

Oncologists in our study were concerned with many factors beyond the minimum regulatory requirements of EA programs. They required that requests for investigational drugs through EA have a clear scientific rationale, a strong ratio of risks to benefits, and a solid safety profile. They also expected patients to have good functional status and high motivation to pursue EA. This rationale meets current regulatory standards that require a patient have a qualifying condition and not be eligible for clinical trials, but also goes beyond them. Together with our participants’ prioritization of functional status over the terminal nature of their patients’ conditions, this rationale provides important nuance around the factors physicians may use to qualify patients to receive unapproved drugs and suggests substantial differences between regulatory and clinical standards for making unapproved therapies available to patients. Raus has analyzed common justifications for compassionate use programs, arguing that most are grounded in justice, autonomy, or beneficence [33]. While law and policy tend to prioritize self-determination and fairness, oncologists in our study appear to more heavily favor beneficence, ensuring that the decision to pursue these pathways is the option they believe to be best for their patient. Further research into how oncologists and other specialists weigh the factors for offering EA to patients would help in understanding and improving access to experimental drugs for patients outside of clinical trials.

The inclusion of patient motivation as part of the rationale oncologists used to offer EA is interesting given their discussion of the low potential of these drugs for benefit and the importance of managing patient expectations. Many scholars differ in their views as to whether physicians should offer experimental options in addition to all reasonable standard of care for every patient. Recognizing the threat of promoting unrealistic optimism of drugs with no potential benefit, Bunnik and Aarts have argued that patient reported factors including affordability, health literacy, or motivation should not guide a physician’s decision when considering whether to discuss experimental interventions [34]. They separately describe regulatory and ideological shifts toward investigational drugs becoming an expectation in clinical settings, raising concerns about false hope, side effects, and funding challenges [35]. Our study shows evidence of high hopes among patients and the powerful role that patient motivation can play, even in contexts where physicians do not believe a particular drug to be helpful for a patient. While affirming the importance of managing expectations, our findings suggest that patient motivations play a complicated role in justifying compassionate use requests, and further research should explore possible differences between patients’ and physicians’ mindsets in considering compassionate use and clinical trial medications while also continuing to engage with broad ethical analysis.

In our study, oncologists reported that patients’ expectations ranged from realistic outlooks to high hopes about experimental interventions, while oncologists themselves considered experimental drugs obtained through EA as mostly palliative. Patients self-reports have also been shown to vary in terms of their motivation to undertake experimental treatment and their expectations of benefit [34]. The extent of a realistic expectation of benefit from experimental products is likely to be based on the patient’s severity of prognosis, despair, frame, risk behavior, and their social interactions with families and caregivers. Unrealistic expectations may add significant burdens on the physicians correcting these beliefs, and this calls into question whether the requirements of informed consent can be upheld. It remains unclear whether physicians ought to intervene, and if so to what extent, especially when considering denying experimental interventions to out-of-option patients who may have high hopes or are unrealistically optimistic [36]. Bunnik et al. call for more explicit investigational drug guidelines, especially in systems that may be shifting toward expanded access as a default, in order to proactively engage with these thorny ethical questions [35].

Conversations in cancer care are inherently difficult given the psychosocial distress on patients and the difficulty of having end-of-life conversations for physicians [37–41]. Our data suggest that oncologists’ views toward patient motivations and expectations of benefit from experimental oncology drugs may differ from what other studies have shown examining the attitudes of patients. While 54% of U.S. adults favor greater access to experimental treatments prior to trials that demonstrate safety and efficacy [42], only 3–5% of patients are enrolled in cancer clinical trials [43], with significantly fewer patients receiving experimental interventions through EA. The source of these disparate numbers suggests that patients may desire access to experimental drugs but perhaps outside of a clinical trial. While therapeutic misconception (the concept that patients may conflate the goals of a clinical trial with the goals of care) exists among oncology patients, adequate informed consent processes shows that patients become less interested in accessing experimental medication if it means they will be randomized and could be placed in a placebo or even a standard of care arm. These numbers also suggest a potentially large disconnect between what patients theoretically may want versus the realities of experimental medicine where toxicities are overwhelming and the idea of taking novel medicine that could help is no longer theoretical.

Our study has some limitations. As a qualitative interview study, our results are not intended to be generalizable. This study was performed at a single institution with robust support for clinical trials, administrative assistance for completing EA requests, and extensive physician experience with EA programs. Our results are further limited as the Mayo Clinic Cancer Center may not necessarily reflect other oncology settings. Finally, our findings are only reflective of physicians’ views and only include interpretations of patients’ perspectives. Further research is needed with patients themselves to share their own interpretations of similar encounters.

## Conclusion

Our study highlights that oncologists at a single medical center with three major locations across the U.S., all of whom have at least some experience with EA and most with research experience, are supportive of EA programs and are willing to support patients who seek experimental treatments outside of clinical trials. Despite our conservative selection of participants with EA experience, several oncologists still experienced hardships and some were unclear about terminology and how best to navigate the multi-stakeholder processes. This suggests that oncologists considering non-trial experimental treatments for patients could benefit from training about preapproval access and need institutional infrastructure to help navigate the application processes. The results from this study also demonstrate that oncologists’ rationales for considering experimental drugs for their patients differ significantly from regulatory standards.

Tools designed to enhance shared decision-making and facilitate conversations about both on- and off-trial medications with patients are needed, and may bridge the gap among discordant views between patient and oncologist [44]. Both physicians and patients are confused with terminology and processes surrounding non-trial preapproval pathways suggesting a need for physician education [1, 26, 34]. While internet databases, social media support groups, and patient advocacy organizations have been highlighted as potential avenues of information about accessing experimental drugs for patients [45], there remains little information among these sources about accessing non-trial experimental treatments compared to information about clinical trials [46]. Tools to foster dialogue between patient and oncologist and support systems for oncologists to navigate non-trial EA are needed so that patients can receive the best care at a crucial juncture late in their illness.

## Supporting information

S1 Interview guide (DOCX)
