# Supplementary material for: Oncologists’ reflections on patient rights and access to compassionate use drugs: A qualitative interview study from an academic cancer center
Source: PLoS One. 2021 Dec 17;16(12):e0261478. doi: 10.1371/journal.pone.0261478 (PMC8682887; doi:10.1371/journal.pone.0261478)
Supplement: S1 Interview guide — (DOCX) [file pone.0261478.s001.docx]

**S1. Interview Guide**

This supplemental section includes the interview guide for the study by Stout et al., “Oncologists’ reflections on patient rights and access to compassionate use drugs: a qualitative interview study from an academic cancer center” *PLOS One*. The interview guide was originally published in Smith et al., (2021) “‘I think it’s been met with a shrug:’ Oncologists’ views toward and experiences with Right-to-Try.” *Journal of the National Cancer Institute* 113(6): 735-741. doi:10.1093/jnci/djaa137

**Interview Guide**

**Part 1**

**Thank you for your willingness to participate in an interview today. We appreciate your time.** We have a short survey to complete before we begin the interview. This will help us determine which questions to ask and which ones to skip. While we look forward to hearing more about your specific experiences later in the interview, for this portion we are looking for numerical or yes or no answers. If you need any clarification about questions, please let me know.

1. From a scale of 1 to 5 where 1 is lowest and 5 is highest, how would you rate your familiarity with the FDA’s Expanded Access Program?

Score: 1-5 points

2 Please answer Yes or No to the following questions surrounding your experience with expanded access:

(a) Have you ever had a conversation with a patient about accessing experimental drugs through expanded access? If Yes, answer (b) through (f).

If you answered Yes to 2(a),

(b) Have you ever requested an experimental drug through expanded access from a sponsor on behalf of a patient?

(c) Have you ever submitted an expanded access request to your Institutional Review Board for ethics review?

(d) Have you ever submitted an expanded access request to the FDA?

(e) After receiving the necessary approvals, have you ever administered informed consent to a patient and administered the experimental drug to the patient?

(f) Have you collected patient outcomes data after administering the experimental drug to report to regulators and/or sponsors?

3. From a scale of 1 to 5 where 1 is lowest and 5 is highest, how would you rate your familiarity with the federal or state Right to Try laws?

4. Please answer Yes or No to the following questions on Right to Try:

(a) Have you ever had a conversation with a patient about accessing experimental drugs through Right to Try legislation?

If you answered Yes to 4(a),

(b) Have you ever requested an experimental drug through Right to Try from a sponsor on behalf of a patient?

(c) Have you ever submitted a Right to Try request to your Institutional Review Board for review?

**Part 2**

Now we will transition to the conversational part of the interview. I will ask some questions with potential follow-up questions. Please feel free to elaborate on your answers and explain each response you have.

**(A) Demographic**

**(i) How many years have you practiced medicine (including residency and fellowship training)?**

- Qualifications (medical degree + specialty training + cancer type(s) of interest) from website.

**(ii) Can you provide the distribution of your time between clinical care, administrative, research and other duties?**

**(iii) Are you currently involved as a researcher in any clinical trials?**

*Prompt*

- *What phases?*
- *In what capacity?*

**(B) Knowledge and Familiarity with EAP_ Charged Environments**

**1) Can you briefly explain why you rated yourself a ____ in regard to EAP knowledge?**

*Prompt*

- *Obtain sources of knowledge e.g., popular press, journal articles, institutional guidelines, 21CFR regs.*

**2) What do you think is the likelihood of receiving access to an unapproved drug for your patient?**

*Prompt*

- *Capture factors that participant perceives to influence the likelihood of access?*

**(C) Patient-Physician Conversations About Unapproved Medications**

**1) In general, who has brought up the conversation about unapproved or experimental treatments outside of clinical trials?**

*Prompt*

- *How does the conversation come about?*
- *If by patient, how is the topic approached?*
- *If by the provider, how do you decide whether an unproven treatment is appropriate for the patient? E.g., what factors influence your decision i.e., likelihood of helping the patient, patient/family desire.*

**2) Please describe a memorable encounter or a specific case discussing with your patient the potential of accessing an unapproved treatment via EAP or RTT (not a clinical trial).**

*Prompt*

- *What was discussed? What do patients ask?*
- *How long had you been treating this patient?*
- *What other options had the patient attempted?*
- *What is your impression of the patients’ comprehension of EAP and RTT?*

**3) Please describe uncertainty—or lack thereof—felt by you or the patient in considering these experimental treatments.**

*Prompt*

- *Describe any unanswered questions about the risk or benefit of the intervention.*
- *How did you engage in this conversation with especially vulnerable patients (or families)?*
- *What were your experiences in providing informed consent?*

**4) How would you rate your level of comfort having conversations about unapproved/experimental treatments with your patients?**

*Prompt*

- *See if there is an association between low EAP experience or less research experience with comfortability.*

**5) Did you feel any ethical or moral tensions when trying to make this decision?**

*Prompt*

- Can you describe any thoughts, feelings, or emotions—either comfortable or uncomfortable—associated with this conversation or process?

**6) What do you think patients are looking for when they request experimental medications?**

*Prompt*

- *Patients are looking for hope, an additional option, a little more time?*
- *Patients desire “control” over their condition and do not wish to be passive?*

**7) How do you manage patient and family expectations?**

*Prompt*

- *Was it difficult to manage patient expectations or family’s expectations?*
- *Do patients/families expect the unproven treatment to help/cure?*

**8) How did you identify potential non-trial experimental treatments for your patient?**

*Prompt*

*Did you check NIH website, ClinicalTrials.gov, other resources?*

**(D) Consenting Patients *(For participants who had already administered drug to patient)***

**1) How did you describe the risks and benefits of the experimental drug?**

*Prompt*

- *Do you think this well understood by the patient?*
- *How is this similar or different to informed consent for regular clinical trials?*

**2) What do you think is the extent of a patient’s understanding about the experimental medication?**

*Prompt*

- *Good/Poor. Did the patient seem well-informed about the experimental nature of the intervention (gauge therapeutic misconception)?*
- *What about family members?*

**(E) Oncologists’ Experience with EAP**

**1) Using a scale of 1-5 where 1 is mostly negative and 5 is mostly positive, how would you rate your overall experience with the EAP?**

*Considerations*

*• Requesting drugs to manufacturers*

*• Filing applications to FDA, IRB*

*• Providing treatment*

*• Reporting outcomes*

**2) Remembering the initial time you completed an EA request, do you recall which resources you found helpful? Consider both external and institutional resources.**

*Prompt*

*• Specifically probe about internal Mayo Clinic resources*

*Tease out possible areas of frustration with external and internal resources*

*• External Resources: FDA webpages; Reagan-Udall Foundation Expanded Access Navigator – Physician; ASCO University’s Expanded Access Series courses*

*• Internal (Institutional) Support/Resources: including go-to person at IRB, help complete FDA application, how to approach manufacturer.*

**3) Did you find any part of the process burdensome? And if so, what kind of burdens did you experience?**

*Considerations/Prompt*

*• How long did the process take total? Hours of input from physician and from staff?*

- *Get participant to consider her/his time and that of assistants/staff*

*• What part of the process (if any) was burdensome? – manufacturer requests, IRB submission/approval, FDA submission/approval, obtaining the drug, managing family expectations*

*• Were the burdens due to lack of standardization of processes?*

*• Were the burdens due to a lack of a clear resource or person to help navigate?*

**(F) Assessing Familiarity with Right to Try**

**1) In the previous survey, we asked you to rate your familiarity with RTT on a scale of 1 to 5 where 1 was lowest and 5 was highest. You reported you were a ____. Can you please explain why?**

*Prompt*

- *What do you think is the difference between EAP and RTT if you had to explain it to someone else?*
- *Why do you think RTT came about? (Patient-led, politics, etc.)*
- *Obtain sources of knowledge e.g., popular press, journal articles, institutional guidelines, federal RTT Law, MN/TX RTT law.*
- *Identify whether they understand that RTT is really a right to ask to obtain an experimental drug and not a new right for patients to access experimental drugs.*
- *Do they know any provisions of MN/TX RTT laws or the federal RTT law?*
- *Can they explain differences between the federal RTT law and EAP e.g., latter requires FDA review, IRB review, both require consent of patient, EAP is for serious diseases/immediately life threatening condition whereas federal RTT is only life threatening conditions, EAP is any stage whereas RTT is after P1.*

**Offer Explanation of EAP vs Federal RTT**

***The following explanation was offered to those individuals who conflated EAP and RTT or explained they did not know or had heard of RTT.***

**Explain:** 1) the federal RTT law permits physicians to request investigational drugs from sponsors after they pass Phase 1 testing and are under an active IND; 2) RTT does not require FDA involvement; and 3) RTT does not require IRB review.

**1) Have you ever had a conversation with a patient about RTT? If so, will you tell me more about that experience?**

**(G) Ethical and Professional Obligations Under EAP/RTT**

**1) Should terminally ill patients be permitted to try any treatment they wish? Why/Why not?**

**2) What do you believe are the main ethical considerations when pursuing an experimental intervention for your patient?**

*Prompt*

- *Tease out the differences between ethical considerations under RTT and EAP.*
- *With experienced participants, tease out whether P1 safety is sufficient for prescribing drugs to terminally-ill patients under RTT.*
- *How do you balance an ethical obligation to ensure trials continue versus providing treatment to a terminally-ill patient?*
- *What criteria do you use to permit patient eligibility in terms of unable to participate and exhausting all other options.*

**3) What do you believe are the professional responsibilities of oncologists to patients when it comes to considerations of requesting non-trial experimental medication?**

*Prompt*

- *Do you see navigating patients to experimental options outside of clinical trials as an “ordinary or extraordinary” task? (Something “above and beyond” or something “expected?”*
- *Do you think physicians are obligated to consider experimental medications for patients?*
- *If yes, what does “consideration” mean in terms of practice e.g., should physicians do searches for medications, refer to physicians with experience, undertake the entire process?*

**4) Do you think an oncologist’s professional responsibilities would be the same or different under EAP and RTT?**

*Prompt*

- *Tease out the differences (if any) between perceived professional obligations under RTT and EAP*

**5) Do you anticipate that RTT may impact expectations of patients regarding access to experimental therapies?**

*Prompt*

*If so, do your foresee any challenges or opportunities that are different under this framework?*

**Is there anything else that you would like to add?**

**Thank you again for taking the time. If you have any additional questions about the study, please do not hesitate to reach out to us.**
